# Advances in Screening, Immunotherapy, Targeted Agents, and Precision Surgery in Cervical Cancer: A Comprehensive Clinical Review (2018–2025)

**DOI:** 10.3390/curroncol33010048

**Published:** 2026-01-15

**Authors:** Priyanka Nagdev, Mythri Chittilla

**Affiliations:** UNC Health Blue Ridge, Morganton, NC 28655, USA; mythri.chittilla@unchealth.unc.edu

**Keywords:** cervical cancer, chemoradiation, immunotherapy, pembrolizumab, ADCs, HPV, biomarkers, precision oncology

## Abstract

Cervical cancer remains a significant global health burden, disproportionately affecting women in low- and middle-income countries despite being preventable. Since 2018, rapid advances in molecular profiling, immunotherapy, refinement of minimally invasive surgery, and targeted therapeutics have transformed diagnostic and therapeutic paradigms. This narrative review synthesizes clinical and translational progress across the continuum of care from 2018 to 2025. We summarize the evolving landscape of precision screening—including HPV genotyping, DNA methylation assays, liquid biopsy, and AI-assisted cytology—and discuss their implications for global elimination goals. Surgical management has shifted toward evidence-based de-escalation with data from SHAPE, ConCerv, and ongoing RACC informing fertility preservation and minimally invasive approaches. For locally advanced disease, KEYNOTE-A18 establishes pembrolizumab plus chemoradiation as a new curative standard, while INTERLACE underscores the benefit of induction chemotherapy. In the metastatic setting, survival outcomes have improved with the integration of checkpoint inhibitors (KEYNOTE-826, BEATcc, EMPOWER-Cervical 1), vascular-targeted therapies, and antibody–drug conjugates, including tisotumab vedotin and emerging HER2 and TROP-2–directed agents. We further highlight emerging biomarkers—PD-L1, TMB, MSI status, HPV integration patterns, APOBEC signatures, methylation classifiers, ctHPV-DNA—and their evolving role in treatment selection and surveillance. Future directions include neoadjuvant checkpoint inhibition, PARP-IO combinations, HER3-directed ADCs, DDR-targeted radiosensitizers, HPV-specific cellular therapies, and AI-integrated precision medicine. Collectively, these advances are reshaping cervical cancer care toward biologically individualized, globally implementable strategies capable of accelerating WHO elimination targets.

## Simple Summary

Cervical cancer care treatment has rapidly evolved over the past decade. Personalized therapy, as opposed to one-size-fits-all, has revolutionized cervical cancer. In this review, we summarize key advances that further shape precision management, including biomarkers to guide therapy, personalized surgical treatment, and the expanding use of immunotherapy and targeted agents. We describe how tumor genomics, PD-L1 expression, HPV-related pathways, and emerging molecular signatures are changing treatment selection and improving patient outcomes. The goal of this review is to provide a clear and up-to-date overview of how precision medicine is transforming the diagnosis, treatment, and future clinical direction of cervical cancer.

## 1. Introduction

Cervical cancer remains one of the most preventable yet persistent causes of cancer mortality among women worldwide. Human papillomavirus (HPV) infection is the primary etiologic agent, with persistent high-risk subtypes 16 and 18 accounting for approximately 70% of all cases. In 2022, GLOBOCAN estimated 662,000 new cases and 349,000 deaths, with a mortality rate of 7.1 per 100,000. More than 70% of the population occurs in low- and middle-income countries. These disparities reflect differences in HPV prevalence as well as unequal access to organized screening, vaccination programs, and timely treatment.

Consequently, cervical cancer is widely considered a preventable malignancy, aligning with the World Health Organization’s global elimination initiative targeting an incidence of fewer than four cases per 100,000 women annually [1].

Cervical squamous cell carcinoma (CSCC) and cervical adenocarcinoma (CAdC) are the two major histopathological types, accounting for 85% and 10–12% of all cervical cancers, respectively. Although the natural progression from HPV infection to precancer and invasive carcinoma is well characterized, co-factors such as HIV infection, tobacco use, early sexual debut, and long-term hormonal contraception influence persistence and transformation [2].

In addition to traditional cytology and HPV DNA testing, several adjunct diagnostic technologies have been evaluated for the detection of cervical intraepithelial neoplasia. These include optoelectronic and colposcopy-enhancing tools such as TruScreen, (TruScreen Group Ltd., Auckland, New Zealand) ZedScan, (Zilico Ltd., Manchester, UK) and DySIS (DYSIS Medical Ltd., Edinburgh, UK). These methods utilize real-time electrical or optical properties of tissues to enhance the identification of precancerous lesions. As HPV-based screening programs become more widespread, effective triage of HPV-positive individuals is increasingly essential. Early real-world assessments of optoelectronic techniques have demonstrated moderate diagnostic accuracy, prompting renewed interest in prevention strategies that rely on HPV detection [3].

Preventive HPV vaccination has further reshaped the epidemiology of cervical cancer by reducing population-level HPV prevalence and promoting viral clearance in vaccinated cohorts. Longitudinal genotyping and phenotyping studies indicate a declining relative contribution of HPV genotype 18, while persistent circulation of other high-risk genotypes has informed updated vaccination strategies. Consequently, many scientific societies now preferentially recommend the nine-valent HPV vaccine, which provides broader genotype coverage and aligns with contemporary shifts in HPV genotype distribution. Integration of HPV genotyping and molecular phenotyping into screening and diagnostic pathways enhances risk stratification and supports personalized surveillance and management [4].

Over the past decade, cervical cancer management has shifted from a uniform, stage-based model to an increasingly personalized approach. Precision screening tools—including DNA methylation assays, HPV integration detection, circulating HPV DNA, and artificial intelligence–assisted cytology complement traditional HPV DNA testing and cytology. Therapeutic strategies aimed at eradicating HPV and reversing precancerous lesions have been refined as pivotal measures for disease prevention. In early-stage disease, contemporary trials such as SHAPE and ongoing investigations like RACC inform safer pathways for conservative or minimally invasive surgery. For locally advanced disease, the integration of immune checkpoint inhibition with chemoradiation—most notably in the KEYNOTE-A18 trial—represents a significant shift in curative-intent therapy [5]. In the recurrent and metastatic setting, the emergence of PD-1 blockade, antibody–drug conjugates, and HER2- and TROP-2–directed therapies has transformed systemic treatment options. Increasingly, biomarkers such as PD-L1 expression, MSI status, tumor mutational burden, HPV genotype, and circulating HPV DNA guide therapeutic decision making.

This narrative review synthesizes key clinical and translational advances from 2018 to 2025, highlighting innovations in screening, surgical de-escalation, immuno-oncology, targeted therapies, and biomarker-driven personalization. Future directions and implementation challenges are discussed in the context of achieving equitable, precision-based cervical cancer care.

## 2. From HPV Infection to Malignant Transformation: Molecular Underpinnings of Cervical Cancer

High-risk human papillomavirus (HPV) initiates a multistep process that progresses from viral infection to precancer and invasive carcinoma over 10–15 years. HPV infects basal epithelial keratinocytes, and viral genome integration into host DNA is a critical event in malignant transformation. Following integration, the constitutive expression of the viral oncoproteins E6 and E7 disrupts cell-cycle regulation by inactivating the p53 and retinoblastoma (Rb) pathways, thereby enabling unchecked proliferation, impaired apoptosis, and genomic instability.

In addition to E6 and E7, the HPV E5 oncoprotein contributes to early stages of cervical carcinogenesis by enhancing epidermal growth factor receptor (EGFR) signaling, disrupting endosomal acidification, and impairing antigen presentation through downregulation of MHC class I molecules.

As a virally driven tumor, cervical cancer shows notably higher lymphocyte infiltration compared to HPV-negative malignancies. Moreover, the increased presence of CD8+ tumor-infiltrating lymphocytes (TILs) has been linked to improved survival rates. HPV-driven tumors exhibit impaired antigen presentation and reduced interferon signaling, which contribute to immune escape and support the rationale for checkpoint blockade. PD-L1 expression appears to play a significant role in establishing an ‘immune-privileged’ site for the initiation and persistence of HPV infection by downregulating T-cell activity and inducing adaptive immune resistance. Its expression has not been demonstrated in normal cervical tissue; however, it is detectable in 95% of cervical intraepithelial neoplasia cases and in cervical cancer T cells, antigen-presenting cells (APCs), and tumor cells [6].

A landmark multi-omic analysis by The Cancer Genome Atlas (TCGA) provided a comprehensive molecular characterization of cervical cancer, identifying APOBEC-driven mutagenesis as a dominant process and recurrent alterations in *PIK3CA*,* PTEN*,* KRAS*,* ERBB3*,* CASP8*,* HLA-A*,* SHKBP1*, and *TGFBR2*.

Copy number analyses further revealed amplifications involving the immune checkpoint ligands CD274 (PD-L1) and PDCD1LG2 (PD-L2), as well as the long non-coding RNA *BCAR4*, which has been associated with lapatinib responsiveness [7].

Importantly, integrative clustering identified three biological subtypes—keratin-low squamous, keratin-high squamous, and adenocarcinoma-rich—highlighting heterogeneity among HPV-driven tumors and suggesting future subtype-directed therapeutic strategies [7]. Alterations in the PI3K/AKT/mTOR pathway are prevalent, with *PIK3CA* mutations found in ~35% of adenocarcinomas and ~25% of squamous cancers [8]. These promote oncogenic PI3Kα activation, proliferation, and therapeutic resistance, forming the rationale for PI3Kα inhibitors such as alpelisib in *PIK3CA*-mutant disease.

*KRAS* mutations are more prevalent in adenocarcinoma associated with advanced FIGO stage, distant metastases, and worse recurrence-free survival. *KRAS*-mutant tumors show a strong correlation with HPV-18 infection. Preclinical data suggest that *KRAS*-driven signaling may contribute to radio resistance in cervical cancer, although its clinical relevance remains to be fully established. The combined assessment of *KRAS* mutation and HPV genotype may help identify patients at high risk of poor prognosis, thereby guiding subsequent therapeutic interventions [9].

Other recurrent alterations include,

*ARID1A* loss: driving YAP1-mediated proliferation [10].
*PTEN* loss promotes tumor progression and apoptosis resistance in cervical cancer and may represent a future therapeutic target [11].

The integration of these biological features underpins contemporary biomarker-driven and precision oncology strategies.

## 3. Immunologic and Biologic Biomarkers in Cervical Cancer

Cervical cancer is biologically heterogeneous, and patients treated with similar therapeutic regimens often exhibit markedly different responses and outcomes. This variability is driven by differences in tumor genomics, immune-microenvironment composition, and host systemic immunity. Consequently, identifying biomarkers that can distinguish future responders from non-responders to chemoradiotherapy remains a significant research priority, with the potential to improve outcomes while reducing overtreatment and healthcare costs [12].

### 3.1. Tumor Microenvironment and Immune Escape

The cervical cancer tumor microenvironment (TME) is characterized by pronounced immunosuppression. Regulatory T cells (Tregs) and myeloid-derived suppressor cells (MDSCs) inhibit cytotoxic T-cell activity by secreting immunosuppressive mediators, such as TGF-β and IDO. Tumor-infiltrating lymphocytes (TILs), although present in high density due to viral antigenicity, often become exhausted or dysfunctional, diminishing anti-tumor immunity. Stromal fibroblasts and endothelial cells further contribute to progression by secreting pro-angiogenic and pro-inflammatory factors that promote survival, invasion, and metastasis.

### 3.2. Other Immune Checkpoints: CTLA-4, LAG-3, and Emerging Targets

CTLA-4 suppresses early T-cell activation, and its upregulation contributes to immune evasion; combination PD-1 + CTLA-4 blockade has demonstrated synergistic immune activation in early trials. LAG-3 is highly expressed in TILs within HPV-driven tumors and contributes to T-cell exhaustion, making it an attractive emerging target. Persistent HPV infection also drives TGF-β signaling, which promotes Treg expansion, impairs cytotoxic T-cell activity, and facilitates epithelial-to-mesenchymal transition, invasion, and metastasis [13].

### 3.3. Genomic Biomarkers: MSI, TMB, HPV Integration, and APOBEC Mutagenesis

Microsatellite instability–high (MSI-H) is uncommon in cervical cancer (≈2–4%, predominantly adenocarcinoma) but carries substantial therapeutic relevance. MSI-H tumors exhibit high neoantigen loads and robust CD8^+^ infiltration, rendering them highly responsive to PD-1 blockade. In the KEYNOTE-158 basket study, pembrolizumab achieved ~30% durable responses in MSI-H/dMMR tumors, including cervical cancer [14]. Key biomarkers used in the precision management of cervical cancer are summarized in Table 1.

Tumor mutational burden (TMB) in cervical cancer typically ranges from 5–10 mut/Mb, primarily driven by APOBEC-mediated mutagenesis. TMB-high tumors may be more responsive to immunotherapy. Emerging evidence suggests that TMB could serve as a predictive biomarker for pembrolizumab in previously treated recurrent or metastatic disease [15].

TCGA multi-omic profiling has identified HPV integration sites, DNA-methylation patterns, and APOBEC signatures as defining genomic features of cervical cancer, revealing three molecularly distinct subtypes with implications for personalized therapy.

### 3.4. Protein and Nucleic Acid Biomarkers

The p16/Ki-67 dual-stain assay is a validated triage tool for CIN2+, while E6/E7 mRNA testing demonstrates high sensitivity for detecting transforming infections [16].

MicroRNAs (miRNAs), dysregulated through methylation changes induced by E6/E7 oncoproteins, are emerging biomarkers for early detection, prognostication, and prediction of chemoradiotherapy resistance [17]. Beyond therapeutic biomarkers, DNA methylation assays are emerging as precision tools for early detection and risk stratification in cervical cancer. HPV-positive women exhibit distinct host gene hypermethylation patterns, and several assays now demonstrate validated clinical performance. The S5 classifier (integrating viral and host gene methylation, including EPB41L3) shows ≈80–90% sensitivity for CIN3+ and superior specificity compared with cytology in HPV-based screening. The FAM19A4/miR124-2 methylation panel, commercially available as QIAsure™, has been validated across multiple European cohorts as a reliable triage for HPV-positive women, including those older than 30 years, for whom cytology sensitivity declines. These methylation signatures provide objective, automatable, and reproducible molecular triage, representing a key step toward precision prevention that complements therapeutic precision oncology strategies [18].

## 4. Clinical Management and Precision Therapy (2018–2025)

### 4.1. Staging Evolution in Cervical Cancer

The 2018 FIGO revision marked a significant shift from a purely clinical staging approach to an imaging- and pathology-informed framework. Integration of MRI, CT, and ^18^F-FDG PET/CT substantially improves assessment of tumor size, depth of stromal invasion, and lymph node metastasis—domains in which clinical examination alone shows discordance in up to 25% of early-stage and nearly 40% of locally advanced cervical cancers. In 2024, FIGO issued updated staging clarifications refining the 2018 revision, particularly regarding tumor size thresholds, nodal designation (r vs. p), and the prioritization of pathological assessment when both imaging and histology are available [19]. The update also clarified that adenocarcinomas follow the same depth-based criteria as squamous carcinomas. LVSI, although prognostically necessary, does not alter FIGO stage and should be reported separately.

### 4.2. Role of Imaging in Modern Cervical Cancer Staging and Treatment Planning

MRI is the preferred modality for evaluating primary tumor characteristics, whereas PET/CT enhances detection of nodal and distant metastases and is recommended by NCCN v.1, 2026, for disease ≥ IB2 [20]. PET-positive nodal disease should be recorded with the “r” modifier in FIGO staging. Prospective trials continue to refine imaging precision: the PRODIGYN trial demonstrated that adding PET/CT or PET/MRI to MRI-based staging resulted in meaningful upstaging and that MRI-derived ADC values and volumetrics correlated with early treatment response [21].

Similarly, a prospective PET/MRI trial is evaluating whether combined metabolic-anatomic imaging improves lymph node detection compared with PET/CT alone, an area of unmet need given that nodal metastasis upstages patients to FIGO IIIC and mandates intensified treatment [22].

Additionally, an earlier prospective study investigating serial MRI and PET during chemoradiation showed that mid-treatment metabolic response predicted progression-free survival, supporting the incorporation of dynamic imaging biomarkers into personalized treatment adaptation [23,24].

These recommendations formalize the transition from clinically based staging to an imaging-driven paradigm, ensuring precise selection for fertility-sparing approaches, surgical vs. chemoradiation pathways, and tailored radiation fields.

### 4.3. Surgical Management of Early-Stage Cervical Cancer

For treatment purposes, cervical cancer is broadly classified into early-stage (FIGO 2018/2024 stages IA1–IB3 and IIA1–IIA2), locally advanced (IIB–IVA), and advanced/metastatic disease (IVB or recurrent). Early-stage tumors are primarily managed surgically, locally advanced cancer is treated with concurrent chemoradiation and brachytherapy, and advanced disease requires systemic therapy.

#### 4.3.1. Transition Toward Precision Surgery

Minimally invasive radical hysterectomy (MIS) was widely adopted based on earlier observational studies demonstrating favorable perioperative outcomes. However, contemporary evidence emphasizes oncologic safety over surgical convenience, shifting the field toward precision surgery—carefully selected patients, accurate staging, optimized radicality, and avoidance of techniques that compromise oncologic control [25].

#### 4.3.2. Stage IA2–Stage IB1 (<2 cm): Evidence Supporting Radical Surgery, Open Approach, and Selective De-Escalation

The LACC Trial: A Watershed Moment [26]

The LACC trial (2018) fundamentally reshaped surgical management for early-stage cervical cancer. In this phase III study of 631 patients with a median follow-up of 4.5 years, disease-free survival (DFS) was significantly lower with MIS (85.0%) than with open surgery (96.0%), yielding an absolute difference of 11.1 percentage points. Overall survival (OS) favored the open approach, with a 4.5-year OS of 96.2% vs. 90.6% Perioperative morbidity, serious adverse events, and patient-reported quality-of-life scores were comparable. Proposed mechanisms include tumor spillage from uterine manipulators, CO_2_ effects, and intracorporeal colpotomy. Together, these findings established open radical hysterectomy as the global standard for IA2–IB1 disease.

#### 4.3.3. MIS in Tumors < 2 cm: Persistent Debate

Despite the findings of the LACC trial, retrospective studies—including SUCCOR and selected institutional cohorts—suggest that minimally invasive surgery may yield comparable oncologic outcomes in chosen rigorously patients with tumors < 2 cm when strict tumor-containment protocols are employed; however, current NCCN and ESGO guidelines continue to favor open radical hysterectomy as the oncologic standard [27].

#### 4.3.4. The RACC Trial

The RACC (GOG-3020) trial is a prospective, international, randomized phase III study evaluating robot-assisted vs. open radical hysterectomy under optimized surgical protocols (no uterine manipulator, closed vaginal cuff). Mature data are anticipated to determine whether modern robotic MIS can safely re-enter standard practice for small-volume IA2–IB1 disease. The study aims to examine whether modern robotic platforms can achieve noninferior 5-year disease-free survival, with secondary endpoints assessing recurrence patterns, perioperative morbidity, and patient-reported quality of life [28].

### 4.4. Surgical De-Escalation and Fertility Preservation (IB1 < 2 cm)

#### 4.4.1. The SHAPE Trial

The SHAPE trial evaluated whether simple hysterectomy could provide oncologic outcomes equivalent to radical hysterectomy in women with small, low-risk cervical cancers. In this international phase III trial, 700 patients were randomized in equal numbers to radical vs. simple hysterectomy. After a median follow-up of 4.5 years, pelvic recurrence at 3 years was extremely low and comparable between groups: 2.17% in the radical hysterectomy arm vs. 2.52% in the simple hysterectomy arm. Importantly, a simple hysterectomy was associated with significantly reduced operative morbidity and faster recovery. The SHAPE trial, therefore, redefined standard practice toward personalized surgical de-escalation [29].

#### 4.4.2. ConCerv Trial Conservative Surgery for Low-Risk IA2–IB1

ConCerv prospectively evaluated conservative and fertility-sparing surgery in low-risk IA2–IB1 tumors (<2 cm, negative LVSI, and negative margins). Among 100 evaluable patients, 44% underwent fertility-sparing conization, 40% conization followed by simple hysterectomy, and 16% nodal staging after inadvertent hysterectomy. With 5% nodal positivity, 2.5% residual disease, and a 2-year recurrence rate of 3.5%, the trial supports conservative, fertility-sparing surgery as a safe, low-recurrence option in rigorously selected low-risk patients [30].

#### 4.4.3. Fertility-Sparing Surgery in Early-Stage Cervical Cancer

For IA1 with LVSI, IA2, and small IB1 tumors (<2 cm), fertility-sparing conization or radical trachelectomy with nodal assessment yields recurrence rates < 5% with favorable live-birth outcomes. ESGO and NCCN restrict fertility-sparing procedures to tumors < 2 cm with no radiologic or pathologic evidence of nodal or parametrial involvement. Extended radical trachelectomy has demonstrated excellent oncologic control in tumors < 2 cm, as supported by large prospective, multi-institutional series [31,32].

#### 4.4.4. Fertility-Sparing Surgery in Larger Tumors (2–4 cm): Emerging Evidence

Fertility preservation remains investigational for 2–4 cm tumors. The NEOCON-F trial evaluates neoadjuvant chemotherapy followed by trachelectomy, with early feasibility but pending oncologic outcomes [33]. Current guidelines do not endorse fertility-sparing surgery in tumors > 2 cm outside clinical trials.

#### 4.4.5. Specialized Surgical Techniques

Nerve-Sparing Radical Hysterectomy

NSRH preserves pelvic autonomic nerves and reduces bladder, bowel, and sexual dysfunction. Meta-analyses confirm oncologic outcomes comparable to those of conventional radical hysterectomy, with substantially improved functional outcomes, supporting its use in specialized centers [34]. ESGO 2023 endorses NSRH in specialized centers with documented expertise, highlighting its role in optimizing quality of life without compromising cancer control.

Similarly, ovarian preservation is now endorsed by ESGO 2023 and NCCN for premenopausal women with early-stage squamous carcinoma, given a <1% risk of ovarian metastasis; it remains controversial for adenocarcinoma due to higher spread risk.

#### 4.4.6. ABRAX Trial: Management of Intraoperative Positive Lymph Nodes

The ABRAX study provided critical prospective evidence on the optimal management of early-stage cervical cancer when lymph-node metastasis is discovered intraoperatively Among 515 patients, with intraoperative positive nodes, DFS (74%), with no significant differences in recurrence (HR 1.154; *p* = 0.446), local relapse (HR 0.836; *p* = 0.557), or overall survival (HR 1.064; *p* = 0.779).

Only higher FIGO stage and tumor size ≥ 4 cm were predictive of poorer outcomes. ABRAX thus supports stopping surgery and transitioning to definitive chemoradiation when nodal metastasis is detected [35].

### 4.5. Precision Nodal Assessment: Sentinel Lymph-Node Mapping

Lymph-node status remains the strongest prognostic determinant in early-stage cervical cancer and dictates adjuvant treatment. Conventional pelvic lymphadenectomy, while accurate, is associated with complications such as lymphocele, lymphedema, and neurovascular injury. SLN mapping using tracers such as indocyanine green (ICG) allows targeted nodal assessment with markedly reduced morbidity.

#### Evidence from Prospective SENTICOL Trials

The SENTICOL I trial provided the first high-quality prospective evidence supporting SLN mapping in early-stage cervical cancer. Among 145 enrolled patients (139 evaluable), dual-tracer mapping identified ≥1 SLN in 97.8% Sensitivity was 92.0% with an NPV of 98.2%. Notably, no false negatives were observed among the 104 patients (76.5%) with bilateral mapping, establishing bilateral detection as the key determinant of accuracy and providing a basis for its selective adoption in international guidelines [36].

The SENTICOL II randomized controlled trial further validated SLNB as a safe alternative to complete pelvic lymph node dissection (PLND). Of 206 patients, 105 were randomized to SLNB alone and 101 to SLNB + PLND. Bilateral SLN detection achieved 96% sensitivity with an 8% false-negative rate, and no false negatives occurred in the SLN + PLND arm. SLNB alone significantly reduced morbidity—lymphatic complications (31.4% vs. 51.5%; *p* = 0.0046) and neurological symptoms (7.8% vs. 20.6%; *p* = 0.01)—with comparable 3-year RFS (92.0% vs. 94.4%). This established SLNB as a safe, morbidity-sparing strategy when bilateral mapping is achieved [37].

SENTICOL III, an ongoing phase III trial (*n* ≈ 950), aims to confirm non-inferiority of SLNB alone vs. SLNB + PLND in IA1 (LVSI+), IA2, and IB1 disease, with 3-year DFS as the primary endpoint. It mandates bilateral mapping, ultra-staging, and centralized pathology. Results will determine whether SLNB can replace PLND as the standard nodal staging procedure [38].

Based on current evidence, NCCN and ESGO endorse SLNB as the preferred method for tumors < 2 cm when bilateral mapping is achieved.

• If mapping fails unilaterally, side-specific lymphadenectomy is required.
• Intraoperative frozen section can guide the need for immediate para-aortic staging or abandonment of fertility-sparing surgery.

Collectively, the SENTICOL trials establish SLNB as an accurate, low-morbidity alternative to complete lymphadenectomy in carefully staged early cervical cancer, reliably detecting nodal disease while reducing long-term complications and supporting modern precision-surgery principles.

## 5. Systemic and Targeted Therapies in Cervical Cancer

The management of cervical cancer has evolved substantially in the past decade, driven by advances in systemic and biomarker-guided therapies. While surgery remains the cornerstone for early-stage disease, most cervical-cancer-related deaths occur in patients with locally advanced or metastatic tumors, in whom systemic therapy constitutes the primary treatment modality. The traditional backbone of cisplatin-based chemoradiation has now been complemented by anti-angiogenic therapy, immune checkpoint inhibition, and antibody–drug conjugates (ADCs), reflecting a broader transition toward personalized, molecularly informed oncology. This section summarizes the evolution of systemic therapy in cervical cancer from 2018 to 2025, highlighting how biomarker-driven regimens and combination approaches are reshaping outcomes.

### 5.1. Concurrent Chemoradiation: The Backbone of Curative Therapy

Concurrent chemoradiation (CCRT) with weekly cisplatin remains the standard curative approach for locally advanced cervical cancer (FIGO IB3–IVA) [39]. Its foundation comes from several landmark randomized trials:

RTOG 90-01 showed that adding concurrent cisplatin-based chemotherapy to pelvic radiation significantly improved overall and progression-free survival vs. radiation alone [40]. GOG 85, GOG 123, and GOG 120 consistently demonstrated superior survival with cisplatin-containing regimens, establishing weekly cisplatin 40 mg/m^2^ as the preferred radiosensitizer [38].

SWOG 8797/GOG 109 demonstrated that adjuvant RT + cisplatin + 5-FU improved 4-year OS (81% vs. 71%) and DFS (80% vs. 63%) vs. RT alone, defining postoperative chemoradiation as the standard for node-positive or margin-positive disease [41].

These pivotal trials have shaped current global guidelines (NCCN v1.2026; ESMO 2024), which recommend EBRT plus weekly cisplatin, followed by image-guided brachytherapy, as the definitive standard.

The definitive regimen consists of external-beam radiation therapy (EBRT) delivered concurrently with weekly cisplatin 40 mg/m^2^, followed by image-guided brachytherapy (IGABT)—a protocol validated by EMBRACE-I and II for achieving durable local control with acceptable toxicity.

The landmark EMBRACE I study evaluated definitive chemoradiotherapy: external-beam radiation with (45–50 Gy in 1.8–2 Gy fractions) with weekly cisplatin for 5–6 cycles followed by MRI-based image-guided adaptive brachytherapy (IGABT). Among the 1341 eligible patients, MRI-based IGABT was performed in 98%. After 51 months of follow-up, 5-year local control was 92%, with limited grade 3–5 toxicity (GU 6.8%, GI 8.5%, vaginal 5.7%, fistula 3.2%), demonstrating excellent tumor control and acceptable morbidity [42].

EMBRACE II is a prospective, multicenter interventional study designed to refine and benchmark the outcomes achieved in RetroEMBRACE and EMBRACE I. It builds on these results by using MRI-guided adaptive Intensity-Modulated Radiation therapy (IMRT) with integrated nodal boosts and modern IGABT techniques. The trial incorporates the most advanced technologies currently available for cervical cancer radiotherapy, including MRI-guided adaptive intensity-modulated radiotherapy (IMRT) with simultaneously integrated nodal boosts and MRI-guided adaptive brachytherapy (IGABT) using interstitial techniques. The study standardizes adaptive contouring, daily IGRT, and chemotherapy to improve tumor control further while reducing toxicity [43]. Preliminary results from the EMBRACE II study, presented by Pötter et al. at ESTRO 2025, demonstrated substantial improvements in disease control and toxicity profiles compared with EMBRACE I. Among 1482 patients treated with IGRT-IMRT, concurrent cisplatin, and MR-IGABT, 3-year local control, pelvic control, and distant control were 93%, 94%, and 93%, respectively. Three- and five-year overall survival rates were 87% and 82%, respectively. Late grade 3–5 morbidity occurred in 8.9%, with only 1% experiencing life-threatening events. Notably, EMBRACE II reported significantly reduced late toxicity and improved outcomes in high-risk subgroups, including T3–T4 and N2 disease (corresponding to FIGO III–IVA). However, these data remain preliminary and have not yet undergone peer review. As such, EMBRACE II should be regarded as an ongoing evaluation of optimized radiotherapy delivery rather than evidence supporting modification of current standards of care, and its results require confirmation through complete peer-reviewed reporting and longer follow-up.

### 5.2. Cytotoxic Intensification Around CCRT

The phase III INTERLACE trial evaluated induction paclitaxel–carboplatin administered for six weeks before definitive chemoradiation in locally advanced cervical cancer [44]. Five hundred patients were randomized to receive carboplatin–paclitaxel for 6 weeks (CCRT) vs. CCRT alone.

At a median follow-up of 67 months, induction therapy significantly improved survival: 5-year PFS, 72% vs. 64% (HR 0.65; *p* = 0.013); and 5-year OS, 80% vs. 72% (HR 0.60; *p* = 0.015). Short-course carboplatin–paclitaxel induction before CCRT improves long-term outcomes and represents a promising strategy for high-risk locally advanced disease [45].

In contrast, the phase III OUTBACK trial evaluated adjuvant carboplatin–paclitaxel after standard chemoradiation and showed no improvement in overall or progression-free survival. Toxicity was higher in the adjuvant arm, reinforcing that consolidation chemotherapy should not be routinely used outside clinical trials [46].

### 5.3. Immunotherapy in Cervical Cancer: Locally Advanced and Metastatic Disease

#### 5.3.1. Locally Advanced Cervical Cancer (FIGO IB3–IVA)

Pembrolizumab + Chemoradiation: KEYNOTE-A18—A New Standard of Care

KEYNOTE-A18 is the first phase III trial to demonstrate a clinically meaningful survival advantage with immunotherapy during curative-intent treatment.

Traditionally, management of locally advanced cervical cancer (LACC) has relied on concurrent chemoradiation (CCRT) with weekly cisplatin. Until recently, no systemic therapy had demonstrated a survival benefit when added to definitive chemoradiation. This changed with the introduction of immune-checkpoint inhibition in the curative setting. The phase III KEYNOTE-A18 trial randomly assigned 1060 patients with newly diagnosed, high-risk locally advanced cervical cancer to receive pembrolizumab or placebo concurrently with standard cisplatin-based chemoradiotherapy followed by up to one year of maintenance therapy. At a median follow-up of 29.9 months, pembrolizumab significantly improved overall survival, with a 36-month OS of 82.6% compared with 74.8% in the placebo group (HR 0.67; 95% CI 0.50–0.90; *p* = 0.004). The disease has been incorporated into NCCN 2026 as a preferred option [47]. Toxicity was higher in the pembrolizumab group with a greater risk difference (≥5%) of any adverse event with respect to thyroid disorders, leukopenia, hypokalemia, and aspartate aminotransferase elevations. Grade ≥ 3 treatment-emergent (75% vs. 69%), grade ≥ 3 treatment-related (67% vs. 61%), and serious treatment-related (17% vs. 12%) adverse events were higher in the pembrolizumab group compared to the placebo group; the most common toxicities were leukopenia, neutropenia, and anemia.

The CALLA phase III trial enrolled 770 women with newly diagnosed, locally advanced cervical cancer (FIGO 2009 stage IB2–IVA) to receive concurrent chemoradiotherapy (CCRT) with or without durvalumab (10 mg/kg q2w during CCRT, then q4w for up to 24 cycles).

At a median follow-up of 18 months, durvalumab did not significantly improve progression-free survival (HR = 0.84; 95% CI, 0.65–1.08; *p* = 0.17).

No new safety signals were observed. These results, presented at ESMO 2022 and subsequently published in The Lancet Oncology (2024), indicate that PD-L1 blockade with durvalumab during and after CCRT does not yet constitute a new standard of care [48].

#### 5.3.2. Recurrent, Persistent, and Metastatic Cervical Cancer

Historically, outcomes for recurrent, persistent, or metastatic cervical cancer were poor, with a median overall survival (OS) of 10–13 months and progression-free survival (PFS) of 5–6 months in the pre-bevacizumab era [49]. Over the past decade, phase III breakthroughs involving anti-angiogenic therapy, immune-checkpoint inhibitors, and antibody–drug conjugates (ADCs) have reshaped systemic treatment. Current NCCN v1.2026 and ESMO 2024 guidelines endorse a hierarchy of platinum doublet ± bevacizumab as first-line therapy, immunotherapy for PD-L1–positive disease, and ADCs for platinum-refractory settings.

The phase III GOG-240 trial established bevacizumab + chemotherapy as the first-line standard for recurrent/persistent/metastatic disease. Adding bevacizumab to paclitaxel–cisplatin or paclitaxel–topotecan improved median OS from 13.3 to 17.0 months (HR 0.71) and increased response rates, without clinically meaningful deterioration in global quality of life, establishing chemo + bevacizumab as the new first-line standard. The hazard ratio for death was 0.71, and the response rate was higher (48% vs. 36%; *p* = 0.008). The benefit of bevacizumab was consistent across backbones and histologies. Bevacizumab is contraindicated in patients with uncontrolled hypertension, recent bleeding or perforation, and carries fistula risk—particularly in previously irradiated pelvis [49].

Major trials evaluating checkpoint inhibitors and targeted therapy are summarized in Table 2.

#### 5.3.3. Immunotherapy in Advanced Cervical Cancer

Persistent and metastatic cervical cancer exhibits an immune-responsive tumor microenvironment characterized by HPV-induced PD-L1 up-regulation and tumor-infiltrating lymphocytes. This provided the biologic rationale for checkpoint inhibition.

The pivotal phase III KEYNOTE-826 trial established pembrolizumab plus platinum–taxane ± bevacizumab as the new first-line standard for PD-L1 CPS ≥ one recurrent, persistent, or metastatic cervical cancer. In the primary analysis (median follow-up = 22 months), pembrolizumab significantly improved progression-free survival (10.4 vs. 8.2 months; HR = 0.62) and overall survival (26.4 vs. 16.8 months; HR = 0.64) compared with placebo. Toxicity was manageable, with grade ≥ 3 events in 82% vs. 75% of patients—predominantly anemia (30%) and neutropenia (12%) [5].

Beyond PD-L1–positive disease, pembrolizumab also carries tumor-agnostic approvals for MSI-H/dMMR and TMB-high tumors, providing an additional therapeutic pathway for this rare molecular subset of cervical cancers.

The phase III BEATcc trial evaluated the addition of atezolizumab to the standard bevacizumab + platinum–taxane regimen with metastatic, persistent, or recurrent cervical cancer. The combination significantly improved median progression-free survival (13.7 vs. 10.4 months; HR 0.62, *p* < 0.0001) and overall survival (32.1 vs. 22.8 months; HR 0.68, *p* = 0.0046) with manageable toxicity [50]. Although BEATcc establishes a highly active IO + VEGF + chemotherapy strategy in the frontline metastatic setting, the regimen does not currently have FDA approval and has not yet been incorporated as a Category 1 preferred option in NCCN v1.2026.

Second-Line Immunotherapy: EMPOWER-Cervical 1

The phase III EMPOWER-Cervical 1 trial established cemiplimab, an anti–PD-1 monoclonal antibody, as the standard of care for patients with recurrent or metastatic cervical cancer that progressed after platinum-based chemotherapy. At a median follow-up of 16 months, median overall survival was 12.0 months with cemiplimab vs. 8.5 months with chemotherapy (HR 0.69; 95% CI 0.56–0.84; *p* < 0.001), and the objective response rate was 16% vs. 6%, respectively. Grade ≥ 3 treatment-related adverse events occurred in 45% of the cemiplimab group and 53% of the chemotherapy group. These results led to FDA approval in 2021 and inclusion in NCCN v1.2026 and ESMO 2024 guidelines as the preferred second-line regimen following progression on platinum-based therapy [51].

In current practice, PD-L1 CPS ≥ one tumors receive 1L pembrolizumab + platinum–taxane ± bevacizumab (KEYNOTE-826), whereas PD-L1–negative or IO-ineligible patients are treated with chemotherapy ± bevacizumab per GOG-240, and cemiplimab is preferred in the post-platinum setting irrespective of PD-L1.

### 5.4. Antibody–Drug Conjugates (ADCs) and Novel Targeted Therapies

The success of immune checkpoint inhibitors has spurred exploration of novel targeted modalities, particularly antibody–drug conjugates (ADCs). ADCs combine tumor-specific monoclonal antibodies with potent cytotoxic payloads, enabling selective drug delivery and reduced systemic toxicity.

#### 5.4.1. Tissue Factor (TF)–Directed ADCs

Tisotumab vedotin-tftv (TV; Tivdak) is a first-in-class ADC targeting tissue factor (TF), a transmembrane glycoprotein abundantly expressed on cervical cancer cells and associated with tumor progression and angiogenesis. TV couples a TF-directed antibody with the microtubule inhibitor monomethyl auristatin E (MMAE) through a cleavable linker, resulting in internalization and intracellular cytotoxic release.

FDA approval in 2021 was based on the phase I/II innovaTV 201 and phase II innovaTV 204 trials, which demonstrated an objective response rate (ORR) of 24% and a disease control rate (DCR) of 72% in previously treated recurrent or metastatic disease, with manageable toxicity [52].

Confirmatory evidence came from the phase III innovaTV 301/ENGOT-cx12/GOG-3057 trial, which randomized 502 patients with recurrent or metastatic cervical cancer that had progressed after platinum-based doublet chemotherapy. At a median follow-up of 10.8 months (95% CI 10.3–11.6), TV reduced the risk of death by 30% compared with investigator’s-choice chemotherapy (median OS 11.5 vs. 9.5 months; HR 0.70; 95% CI 0.54–0.89; *p* = 0.0038) [42,43]. The 12-month OS rates were 48.7% vs. 35.3%, respectively. Median progression-free survival (PFS) was 4.2 vs. 2.9 months (HR 0.67; 95% CI 0.54–0.82; *p* < 0.0001 [53]. Treatment was generally well tolerated; ocular events (46%), peripheral neuropathy (30%), and epistaxis (26%) were the most frequent adverse events and were mitigated by eye-care prophylaxis and dose modifications. These results, presented at the 2023 ESMO Congress, established TV as a preferred second- or third-line standard for recurrent or metastatic cervical cancer, a finding now reflected in the NCCN v1.2026 and ESGO 2024 guidelines.

#### 5.4.2. Combination Strategies and Emerging ADCs

Interim data from the ongoing innovaTV 205 trials suggest potential synergy between ADCs and immune or platinum-based regimens. Reported ORRs were 54.5% with first-line TV + carboplatin, 40.6% with TV + pembrolizumab, and 35.3% with second- or third-line TV + pembrolizumab [52]. Further follow-up will determine durability and confirm safety profiles [54].

#### 5.4.3. HER2-Directed ADCs

HER2 overexpression or amplification is found in ~3–6% of cervical adenocarcinomas and <2% of squamous tumors but predicts aggressive biology and potential trastuzumab responsiveness. HER2-targeted ADCs offer a means of personalized therapy in this biologically distinct subset.

Trastuzumab deruxtecan (T-DXd; DS-8201a) is a HER2-directed ADC comprising a trastuzumab backbone linked to the topoisomerase I payload DXd via a cleavable tetrapeptide linker. With a drug-to-antibody ratio of ≈8:1 and high membrane permeability, T-DXd exerts a robust bystander effect, extending efficacy to HER2-low (IHC 1+/2+, ISH-negative) tumors. The DESTINY-PanTumor02 trial extended its evaluation to multiple HER2-expressing solid tumors, including cervical, endometrial, ovarian, and biliary tract cancers [55].

In this open-label phase II study, 267 patients with HER2-expressing advanced or metastatic tumors received T-DXd after ≥1 prior systemic therapy. The overall ORR was 37.1%, median duration of response 11.3 months, median PFS 6.9 months, and median OS 13.4 months. Notably, in patients with centrally confirmed HER2 IHC 3+ expression, the ORR increased to 61.3%, with a median DOR of 22.1 months, PFS of 11.9 months, and OS of 21.1 months. Grade ≥ 3 drug-related adverse events occurred in 40.8% of patients, including interstitial lung disease (ILD) in 10.5%, with three ILD-related deaths.

These findings demonstrate meaningful and durable responses, particularly in strongly HER2-positive tumors, supporting the tumor-agnostic therapeutic potential of T-DXd across HER2-expressing solid malignancies, including cervical carcinoma.

At present, no cervical-specific clinical data exist for combination HER2 ADCs (e.g., T-DXd + pembrolizumab), and such approaches remain investigational.

#### 5.4.4. TROP-2–Directed Antibody–Drug Conjugates

Trophoblast cell-surface antigen 2 (TROP-2) is a transmembrane glycoprotein involved in cell proliferation and epithelial–mesenchymal transition. It is overexpressed in roughly 40–60% of cervical carcinomas, particularly squamous subtypes, where its expression correlates with tumor invasiveness and adverse prognosis. Because normal squamous epithelium expresses minimal TROP-2, it represents a selective and attractive therapeutic target.

Clinical validation was provided by the phase II EVER-132-003 trial, which enrolled 40 Chinese patients with recurrent or metastatic cervical cancer previously treated with a median of two systemic regimens (68% post-immunotherapy). Sacituzumab govitecan achieved an objective response rate (ORR) of 43% (95% CI, 27–59), a median duration of response of 9.2 months, and a median progression-free survival of 7.1 months (95% CI, 4.2–8.4). Responses were observed in patients previously exposed to immune checkpoint inhibitors (ORR, 48%; median DOR, 9.5 months). Grade ≥ 3 toxicities occurred in 63% of patients. Exploratory analyses indicated no clear correlation between TROP-2 expression intensity and response, suggesting potential benefit across a wide range of expression levels [56].

Treatment options for recurrent, persistent, or metastatic cervical cancer are outlined in Table 3.

## 6. Future Directions

The next decade of cervical cancer research will be defined by precision prevention, biomarker-guided therapy, integrative immunoradiotherapy, and the expansion of targeted and cellular immunotherapies. Advances in multi-omic profiling, circulating tumor DNA (ctDNA), and artificial intelligence are expected to drive individualized treatment strategies across disease stages.

### 6.1. Screening, Prevention, and Early Detection

Global elimination efforts emphasize simultaneous advances in vaccination, screening, and early molecular detection.

HPV FASTER Strategy (Europe ongoing)

The HPV FASTER initiative integrates prophylactic HPV vaccination with HPV DNA screening for women aged 25–45. Early modeling suggests accelerated reductions in cervical cancer incidence, improved cost-effectiveness, and the possibility of longer screening intervals. Ongoing implementation programs in Europe incorporate digital registries and population-level tracking, aligning with WHO 90–70–90 elimination goals [57].

Genotype-Based and Self-Sampling Innovations

The ATHENA trial validated HPV16/18 genotyping as a risk-stratification tool in women aged ≥ 25 years, superior to cytology, supporting its incorporation into precision screening algorithms. Follow-up analyses (Lancet Oncol 2020 updates) reaffirmed the value of extended genotyping in refining screening intervals and guiding colposcopy referral [58]. The VALHUDES study demonstrated high concordance between clinician-collected and self-collected HPV samples, establishing self-sampling as an effective strategy for expanding screening access in underserved or low-resource regions [59].

### 6.2. Immunoradiotherapy and Immune-Priming Approaches

Similarly, tumor mutational burden (TMB) has emerged as a promising biomarker, with high-TMB tumors demonstrating enriched neoantigen landscapes and potentially greater responsiveness to PD-1/PD-L1 blockade [60]. Single-cell mapping of tumor and immune compartments, showing HPV-antigen-specific T-cell signatures, interferon-γ–driven inflammation, and exhaustion markers that correlate with response-relevant immune phenotypes [61] .

Next-generation checkpoint targets (CTLA-4, LAG-3, TIGIT): Immune escape in HPV-driven tumors extends beyond PD-1/PD-L1, underscoring the need for multi-pathway combinations. CTLA-4 blockade (e.g., nivolumab + ipilimumab in CheckMate-358) has shown early evidence of synergistic immune activation. This phase 1/2 trial demonstrated that the combination of nivolumab and ipilimumab achieved durable responses in recurrent or metastatic cervical cancer, suggesting synergy of CTLA-4 plus PD-1 blockade. The recurrent/metastatic cervical cancer cohort of CheckMate 358 evaluated nivolumab alone and nivolumab plus ipilimumab across multiple dosing strategies in previously treated patients. Among 176 treated patients, objective response rates were 26% with nivolumab, 31–40% with nivolumab + ipilimumab, and responses were durable over 12–20 months of follow-up. These results highlight promising dual-checkpoint synergy and justify future randomized trials of PD-1 + CTLA-4 blockade in recurrent/metastatic cervical cancer. Toxicity was manageable overall, although combination therapy produced higher rates of grade ≥ 3 immune-related events and one treatment-related death due to colitis [62].

LAG-3: Overexpression in HPV-driven tumors supports evaluation of LAG-3 inhibitors in combination regimens [63].

TIGIT: Preclinical evidence indicates restoration of CD8^+^ function, with early trials exploring TIGIT + PD-L1 combinations and early phase II trials (e.g., tiragolumab + atezolizumab) are exploring TIGIT inhibition in this setting [64].

PD-1 + VEGF Inhibition: Pembrolizumab + Lenvatinib: Pembrolizumab combined with lenvatinib represents a promising strategy for reversing VEGF-mediated immunosuppression. A phase II trial is testing this combination in locally advanced or metastatic cervical cancer that has progressed after first-line therapy, using Simon’s two-stage design with ORR as the primary endpoint. This approach may enhance immune responsiveness in PD-1–refractory disease and provides a rationale for future randomized trials evaluating VEGF–IO combinations in earlier lines of therapy [65].

#### Neoadjuvant and Adjuvant Immunotherapy

Early-stage high-risk tumors may benefit from immunotherapy earlier in the treatment pathway NRG-GY017 (NCT03738228) demonstrated that neoadjuvant PD-L1 blockade before chemoradiation induces a significantly greater expansion of tumor-associated T-cell receptor clones at day 21 and showed a favorable, though not statistically significant, improvement in 2-year DFS (76% vs. 56%; *p* = 0.28), supporting neoadjuvant immune priming as a feasible strategy for future phase II–III [66].

NRG-GY037 is a forthcoming phase III randomized trial evaluating whether immune-chemotherapy priming can enhance outcomes in high-risk, locally advanced cervical cancer. The trial will compare induction pembrolizumab combined with carboplatin and paclitaxel, followed by standard cisplatin-based chemoradiation with pembrolizumab, vs. chemoradiation with pembrolizumab alone. By enrolling patients with FIGO T3–T4 disease—with or without nodal involvement—NRG-GY037 aims to determine whether upfront integration of immunotherapy and cytotoxic chemotherapy before definitive chemoradiation can further improve progression-free and overall survival beyond the established benefit of pembrolizumab added concurrently to CCRT [67].

### 6.3. PARP Inhibitors in Cervical Cancer Emerging Therapeutic Opportunities

PARP inhibition has emerged as a promising strategy in HPV-driven cervical cancer due to intrinsic defects in DNA-damage response pathways. HPV E6/E7 oncoproteins impair homologous recombination via p53 degradation and replication stress, providing a biologic rationale for PARP-based synthetic lethality. PARP1 overexpression further supports its potential as a therapeutic target, although no validated PARP biomarker yet exists.

Preclinical models show that PARP inhibitors (olaparib, niraparib, rucaparib) enhance radiosensitivity, increase DNA double-strand breaks, and activate cGAS–STING–mediated immune signaling, providing a strong basis for combination approaches with chemoradiation and PD-1/PD-L1 blockade [68].

Early-phase clinical investigation is ongoing.

• NCT04068753: A phase II study is evaluating niraparib plus the PD-1 inhibitor dostarlimab in recurrent or progressive cervical cancer. Patients receive daily niraparib with dostarlimab every 3 weeks (then every 6 weeks) until progression or unacceptable toxicity. The trial primarily assesses the safety and preliminary antitumor activity of combined PARP inhibition and immune checkpoint blockade [69].
• NCT04641728: A multicenter, single-arm phase II trial is evaluating pembrolizumab combined with olaparib in recurrent or metastatic cervical cancer after progression on platinum-based chemotherapy. The study plans to enroll 28 patients to assess the safety and preliminary antitumor activity of PARP inhibition plus PD-1 blockade [70].

### 6.4. HER3-Directed ADCs (Daxibotamab Deruxtecan/HER3-DXd)

HER3 (ERBB3) is overexpressed in a subset of cervical cancers and is associated with PI3K/AKT–mediated resistance, making it an attractive biomarker-selected target. Daxibotamab deruxtecan (HER3-DXd) is a next-generation HER3-directed ADC linking a fully human anti-HER3 antibody to a deruxtecan topoisomerase-I payload. Early clinical activity and manageable toxicity have been demonstrated in HER3-expressing solid tumors, including NSCLC and breast cancer. The ongoing HERTHENA-PanTumor01 phase II trial (NCT06172478) is evaluating HER3-DXd across multiple HER3-positive malignancies. Although cervical cancer is not a dedicated cohort, the tumor-agnostic development of HER3-DXd and the presence of HER3 expression in cervical tumors support future investigation in this population [71].

Although still in early development, HER3-DXd represents a biomarker-selected ADC strategy with potential relevance to recurrent/metastatic cervical cancer, especially in tumors exhibiting HER3 overexpression or PI3K pathway activation.

### 6.5. DDR Pathways and Emerging Radiosensitizers (ATR and WEE1 Inhibitors)

HPV-driven cervical tumors exhibit high replication stress and dependence on ATR–CHK1 and WEE1–CDK1 signaling, providing a rationale for DDR-targeted radiosensitizers. ATR inhibitors (e.g., berzosertib) and WEE1 inhibitors (e.g., adavosertib) have shown radio-sensitizing activity in HPV-associated preclinical models, with cervical cohorts included in ongoing Phase I/II trials combining these agents with cisplatin-based chemoradiation. While cervical-specific efficacy data are not yet available, the strong mechanistic rationale and early safety signals support continued investigation of DDR inhibitors, particularly in combination with immunotherapy and radiation [72].

### 6.6. HPV-Targeted Therapeutic Vaccines and Cellular Therapies

Cellular Immunotherapy

TIL therapy has produced durable responses, even in heavily pretreated HPV-positive cervical cancers. LN-145 (lifileucel) TIL therapy achieved an ORR of ~44% with a median DOR > 12 months in heavily pretreated metastatic cervical cancer, with many responses ongoing, positioning TIL therapy as a leading cellular immunotherapy approach despite current CMC-related delays in regulatory approval [73].

Genetically engineered TCR T cells targeting HPV-16 E6/E7 epitopes show potent activity in early-phase trials, highlighting the therapeutic potential of antigen-specific cellular approaches. E7 TCR-T is a phase II clinical trial to assess the clinical activity of immunotherapy with E7 TCR-T cells for metastatic HPV-associated cancers [74].

HPV Therapeutic Vaccines.

DNA, viral vector, and peptide vaccines targeting HPV E6/E7 antigens are being combined with PD-1 inhibitors to enhance antigen presentation and reverse immune escape. These vaccine–IO platforms represent a promising strategy for earlier disease stages and minimal residual disease contexts [75].

Dynamic biomarkers—including ctHPV-DNA monitoring [76] and TCR sequencing [77]—are increasingly incorporated into trials to identify early responders, and radiomic signatures to guide escalation or de-escalation of therapy [78]. Persistent detection of circulating HPV DNA after completion of chemoradiation is independently associated with significantly worse progression-free survival, and end-of-treatment ctHPV-DNA testing can reliably identify patients at the highest risk of recurrence who may benefit from future treatment intensification or maintenance immunotherapy [79].

### 6.7. Biomarkers, ctDNA, and AI-Driven Precision Care

#### 6.7.1. ctDNA and MRD Surveillance

Personalized ctDNA assays based on HPV integration and mutation profiles show promise for early recurrence detection, real-time treatment monitoring, and the identification of candidates for IO intensification. Persistent ctHPV-DNA following chemoradiation strongly predicts recurrence and poor survival. A prospective cohort evaluating personalized ctDNA assays based on tumor-specific HPV integration and mutation profiles is exploring their utility for early recurrence detection and immunotherapy stratification in advanced cervical cancer. This customized ctDNA approach may enable individualized surveillance and biomarker-driven treatment decisions [80].

#### 6.7.2. Multi-Omic and Spatial Profiling

Integrated genomic, methylation, and APOBEC-mutational signatures are emerging as robust classifiers for risk stratification. Spatial transcriptomics and digital pathology are elucidating the architecture of immune infiltration and stromal barriers, potentially guiding the selection of IO combinations or radiation dose adaptation [81,82].

#### 6.7.3. AI in Screening and Treatment Planning

AI-assisted cytology and automated HPV triage algorithms have shown high diagnostic accuracy in global studies and are being incorporated into WHO/NCCN frameworks. Radiomics-derived imaging signatures may further refine adaptive radiation strategies and personalize systemic therapy [83].

Together, these innovations point toward a future in which treatment intensity, imaging surveillance, and systemic therapy selection are guided by dynamic, biologically informed markers rather than by static clinical stages.

## 7. Conclusions

Molecular characterization has fundamentally advanced the biological understanding of cervical cancer, elucidating recurrent genomic alterations, dysregulated signaling pathways, and immune-modulatory features that extend beyond histology and clinical stage. These insights have provided a framework for developing biomarker-informed therapeutic strategies, particularly in recurrent and metastatic settings, where immunotherapy and targeted approaches are being actively investigated.

Nevertheless, the translation of molecular discoveries into routine clinical practice remains limited. Most precision-based strategies are supported by early-phase evidence, and the clinical utility of many proposed biomarkers requires prospective validation and standardized implementation. Continued progress will depend on the integration of molecular stratification into rigorously designed clinical trials, coupled with pragmatic consideration of feasibility and access, to ensure that advances in precision oncology yield durable and broadly applicable clinical benefits.

## Figures and Tables

**Table 1:** Key biomarkers in cervical cancer.

Biomarker Category	Biomarker	Prevalence in Cervical Cancer	Clinical/Predictive Significance
Genomic	PIK3CA mutations	25–35%	PI3K pathway activation; potential target for PI3K inhibitors (alpelisib)
Genomic	KRAS mutations (HPV18-enriched)	5–10%; adenocarcinoma	Associated with metastasis and worse CRT response, possible role for ERK/RAF inhibitors
Genomic	ARID1A loss	10–15%	YAP1 activation; poor prognosis; emerging target
Genomic	PTEN loss	8–12%	Activates AKT/mTOR; apoptosis resistance
Immune/TME	PD-L1 expression	80–95%	Predictive for pembrolizumab (KEYNOTE-826); hallmark of adaptive immune resistance
Immune/TME	CD8+ TIL density	High in HPV+ tumors	Strong prognostic marker; associated with better immunotherapy response
Immune/TME	LAG-3/TIGIT	Upregulated in exhausted T cells	Supports dual checkpoint blockade approaches
DNA Repair/MSI	MSI-H/dMMR	2–4% (mostly adenocarcinoma)	High response rates to pembrolizumab (KEYNOTE-158)
DNA Repair/MSI	TMB-High	5–8%	FDA tumor-agnostic pembrolizumab eligibility
Viral/HPV	HPV Genotype 16 vs. 18	HPV16 ≈ 55%; HPV18 ≈ 15%	HPV18 → worse prognosis; HPV16 → more immunogenic
Viral/HPV	HPV E6/E7 mRNA	High in CIN2+ and invasive tumors	Detects transforming infection; used for risk stratification
Methylation	FAM19A4/miR-124-2 methylation	Validated for CIN2+ triage	Molecular triage for HPV-positive women
Methylation	S5 methylation classifier	80–90% sensitivity for CIN3+	Automatable, objective risk stratification
Circulating Biomarkers	ctHPV-DNA	Detected in LACC and metastatic disease	Predicts recurrence, MRD, and early treatment failure
Circulating Biomarkers	TCR clonality/expansion	Seen after neoadjuvant IO (NRG-GY017)	Biomarker of immune activation and response

**Table 2:** Key clinical trials in cervical cancer (2018–2025).

Trial	Population	Intervention	Outcome	Impact
KEYNOTE-A18 (2023)	Newly diagnosed LACC (IB2–IVA)	Pembrolizumab + CCRT vs. CCRT	36-mo. OS 82.6% vs. 74.8% (HR 0.67)	New Standard of Care
CALLA (2024)	LACC	Durvalumab + CCRT vs. CCRT	No significant PFS benefit	Not Standard of Care
EMBRACE II (2025)	LACC	MRI-guided IMRT + IGABT	3-yr LC 93%, OS 87%	Benchmark CRT platform
KEYNOTE-826 (2021)	1L recurrent/metastatic	Pembrolizumab + chemo ± bev	OS 26.4 vs. 16.8 mo. (HR 0.64)	1L SOC for PD-L1^+^
EMPOWER-Cervical 1 (2021)	Post-platinum	Cemiplimab vs. chemo	OS 12.0 vs. 8.5 mo. (HR 0.69)	2L SOC
innovaTV-301 (2023)	Post-platinum	Tisotumab vedotin vs. chemo	OS 11.5 vs. 9.5 mo.	2L/3L ADC SOC

**Table 3:** Systemic therapy options for recurrent, persistent, or metastatic cervical cancer (NCCN 2026/ESMO 2024).

Line of Therapy	Regimen	Indication/Biomarker	Evidence
First Line (Preferred)	Platinum–taxane + pembrolizumab ± bevacizumab	PS-L1 CPS ≥ 1	KEYNOTE-826: OS 26.4 vs. 16.8 mo.
	Platinum–taxane ± bevacizumab	PD-L1 negative/IO-ineligible	GOG-240: OS ↑ to 17.0 mo.
	Platinum–taxane + atezolizumab + bevacizumab	PD-L1 positive (investigational)	BEATcc: OS 32.1 vs. 22.8 mo.
After platinum progression	Cemiplimab (PD-1 inhibitor)	No biomarker required	EMPOWER-1: OS 12.0 vs. 8.5 mo.
	Tisotumab vedotin (Tivdak)	TF-expressing tumors (the majority of CC)	innovaTV-301: OS 11.5 vs. 9.5 mo.
HER2-positive tumors	Trastuzumab deruxtecan (T-DXd)	HER2 IHC2+/3+	DESTINY-PanTumor02: ORR 37–61%
TROP-2–positive tumors	Sacituzumab govitecan	No clear biomarker cutoff	EVER-132-003: ORR 43%

## Data Availability

No new data were created or analyzed in the study.

## References

[1] Grau-Bejar J.F., Garcia-Duran C., Garcia-Illescas D., Mirallas O., Oaknin A. (2023). Advances in Immunotherapy for Cervical Cancer. Ther. Adv. Med. Oncol..

[2] Perkins R.B., Wentzensen N., Guido R.S., Schiffman M. (2023). Cervical Cancer Screening: A Review. JAMA.

[3] Pruski D., Kedzia W., Przybylski M., Józefiak A., Kedzia H., Spaczyński M. (2008). Assesment of real optoelectronic method in the detection of cervical intraepithelial neoplasia. Ginekol. Pol..

[4] Joura E.A., Giuliano A.R., Iversen O.-E., Bouchard C., Mao C., Mehlsen J., Moreira E.D., Ngan Y., Petersen L.K., Lazcano-Ponce E. (2015). A 9-Valent HPV Vaccine against Infection and Intraepithelial Neoplasia in Women. N. Engl. J. Med..

[5] Monk B.J., Colombo N., Tewari K.S., Dubot C., Caceres M.V., Hasegawa K., Shapira-Frommer R., Salman P., Yañez E., Gümüş M. (2023). First-Line Pembrolizumab + Chemotherapy Versus Placebo + Chemotherapy for Persistent, Recurrent, or Metastatic Cervical Cancer: Final Overall Survival Results of KEYNOTE-826. J. Clin. Oncol..

[6] Mezache L., Paniccia B., Nyinawabera A., Nuovo G.J. (2015). Enhanced Expression of PD L1 in Cervical Intraepithelial Neoplasia and Cervical Cancers. Mod. Pathol..

[7] (2017). The Cancer Genome Atlas Research Network. Integrated Genomic and Molecular Characterization of Cervical Cancer. Nature.

[8] Voutsadakis I.A. (2021). PI3KCA Mutations in Uterine Cervix Carcinoma. J. Clin. Med..

[9] Peng Y., Yang Q. (2024). Targeting KRAS in Gynecological Malignancies. FASEB J..

[10] Gao F., Li P., Kong X., Song T., Han Q., Zhou S. (2021). ARID1A-Mutated Cervical Cancer Depends on the Activation of YAP Signaling. Front. Biosci.-Landmark.

[11] Shin J.W., Kim S.-H., Yoon J.Y. (2021). PTEN Downregulation Induces Apoptosis and Cell Cycle Arrest in Uterine Cervical Cancer Cells. Exp. Ther. Med..

[12] Rocha Martins P., Luciano Pereira Morais K., de Lima Galdino N.A., Jacauna A., Paula S.O.C., Magalhães W.C.S., Zuccherato L.W., Campos L.S., Salles P.G.O., Gollob K.J. (2023). Linking Tumor Immune Infiltrate and Systemic Immune Mediators to Treatment Response and Prognosis in Advanced Cervical Cancer. Sci. Rep..

[13] Li Y., Deng J., Liu Y., Yu S. (2025). HPV Infection and the Immune Microenvironment in Cervical Cancer. Front. Immunol..

[14] Chung H.C., Ros W., Delord J.-P., Perets R., Italiano A., Shapira-Frommer R., Manzuk L., Piha-Paul S.A., Xu L., Zeigenfuss S. (2019). Efficacy and Safety of Pembrolizumab in Previously Treated Advanced Cervical Cancer: Results From the Phase II KEYNOTE-158 Study. J. Clin. Oncol..

[15] Marabelle A., Fakih M., Lopez J., Shah M., Shapira-Frommer R., Nakagawa K., Chung H.C., Kindler H.L., Lopez-Martin J.A., Miller W.H. (2020). Association of Tumour Mutational Burden with Outcomes in Patients with Advanced Solid Tumours Treated with Pembrolizumab: Prospective Biomarker Analysis of the Multicohort, Open-Label, Phase 2 KEYNOTE-158 Study. Lancet Oncol..

[16] Giorgi Rossi P., Carozzi F., Ronco G., Allia E., Bisanzi S., Gillio-Tos A., De Marco L., Rizzolo R., Gustinucci D., Del Mistro A. (2021). P16/Ki67 and E6/E7 mRNA Accuracy and Prognostic Value in Triaging HPV DNA-Positive Women. J. Natl. Cancer Inst..

[17] Shen S., Zhang S., Liu P., Wang J., Du H. (2020). Potential Role of microRNAs in the Treatment and Diagnosis of Cervical Cancer. Cancer Genet..

[18] Burdier F.R., Waheed D.-N., Nedjai B., Steenbergen R.D.M., Poljak M., Baay M., Vorsters A., Van Keer S. (2024). DNA Methylation as a Triage Tool for Cervical Cancer Screening—A Meeting Report. Prev. Med. Rep..

[19] Saleh M., Virarkar M., Javadi S., Elsherif S.B., de Castro Faria S., Bhosale P. (2020). Cervical Cancer: 2018 Revised International Federation of Gynecology and Obstetrics Staging System and the Role of Imaging. Am. J. Roentgenol..

[20] Isaji Y., Tsuyoshi H., Tsujikawa T., Orisaka M., Okazawa H., Yoshida Y. (2023). Prognostic Value of 18F-FDG PET in Uterine Cervical Cancer Patients with Stage IIICr Allocated by Imaging. Sci. Rep..

[21] Aasa M., Lindquist D., Ottander U., Strandberg S.N. (2025). Primary Staging with 2[18F]-FDG-PET/CT and -PET/MRI and Radiotherapy Response Evaluation with MRI in Uterine Cervical Cancer: An Interim Analysis of a Prospective Clinical Trial. EJNMMI Rep..

[22] Institut de Cancérologie Strasbourg Europe (2024). Contribution of PET/MRI in Locally Advanced Cervical Cancer.

[23] MRI and PET Imaging in Predicting Treatment Response in Patients with Stage IB-IVA Cervical Cancer|Clinical Research Trial Listing. https://www.centerwatch.com/clinical-trials/listings/NCT01992861/mri-and-pet-imaging-in-predicting-treatment-response-in-patients-with-stage-ib-iva-cervical-cancer.

[24] University of Washington (2023). MRI- and PET-Predictive-Assay of Treatment Outcome in Cancer of the Cervix. https://clinicaltrials.gov/study/NCT01992861.

[25] Poddar P., Maheshwari A. (2025). Surgery for Cervical Cancer: Consensus & Controversies. Indian J. Med. Res..

[26] Ramirez P.T., Robledo K.P., Frumovitz M., Pareja R., Ribeiro R., Lopez A., Yan X., Isla D., Moretti R., Bernardini M.Q. (2024). LACC Trial: Final Analysis on Overall Survival Comparing Open Versus Minimally Invasive Radical Hysterectomy for Early-Stage Cervical Cancer. J. Clin. Oncol..

[27] Chacon E., Manzour N., Zanagnolo V., Querleu D., Núñez-Córdoba J.M., Martin-Calvo N., Căpîlna M.E., Fagotti A., Kucukmetin A., Mom C. (2022). SUCCOR Cone Study: Conization before Radical Hysterectomy. Int. J. Gynecol. Cancer.

[28] Falconer H. (2025). Robot-Assisted Approach to Cervical Cancer. https://clinicaltrials.gov/study/NCT03719547.

[29] Plante M., Kwon J.S., Ferguson S., Samouëlian V., Ferron G., Maulard A., de Kroon C., Driel W.V., Tidy J., Williamson K. (2024). Simple versus Radical Hysterectomy in Women with Low-Risk Cervical Cancer. N. Engl. J. Med..

[30] Schmeler K.M., Pareja R., Lopez Blanco A., Humberto Fregnani J., Lopes A., Perrotta M., Tsunoda A.T., Cantú-de-León D.F., Ramondetta L.M., Manchana T. (2021). ConCerv: A Prospective Trial of Conservative Surgery for Low-Risk Early-Stage Cervical Cancer. Int. J. Gynecol. Cancer.

[31] Smith E.S., Moon A.S., O’Hanlon R., Leitao M.M., Sonoda Y., Abu-Rustum N.R., Mueller J.J. (2020). Radical Trachelectomy for the Treatment of Early-Stage Cervical Cancer: A Systematic Review. Obstet. Gynecol..

[32] Kim C.H., Abu-Rustum N.R., Chi D.S., Gardner G.J., Leitao M.M., Carter J., Barakat R.R., Sonoda Y. (2012). Reproductive Outcomes of Patients Undergoing Radical Trachelectomy for Early-Stage Cervical Cancer. Gynecol. Oncol..

[33] Plante M., van Trommel N., Lheureux S., Oza A.M., Wang L., Sikorska K., Ferguson S.E., Han K., Amant F. (2019). FIGO 2018 Stage IB2 (2-4 Cm) Cervical Cancer Treated with Neo-Adjuvant Chemotherapy Followed by Fertility Sparing Surgery (CONTESSA); Neo-Adjuvant Chemotherapy and Conservative Surgery in Cervical Cancer to Preserve Fertility (NEOCON-F). A PMHC, DGOG, GCIG/CCRN and Multicenter Study. Int. J. Gynecol. Cancer.

[34] Lee S.H., Bae J.W., Han M., Cho Y.J., Park J.-W., Oh S.R., Kim S.J., Choe S.Y., Yun J.H., Lee Y. (2020). Efficacy of Nerve-Sparing Radical Hysterectomy vs. Conventional Radical Hysterectomy in Early-Stage Cervical Cancer: A Systematic Review and Meta-Analysis. Mol. Clin. Oncol..

[35] Cibula D., Dostalek L., Hillemanns P., Scambia G., Persson J., Raspagliesi F., Novak Z., Jaeger A., Capilna M.E., Weinberger V. (2020). 806O Radical Hysterectomy in Cervical Cancer Patients with Intraoperatively Detected Positive Lymph Node: ABRAX Multicentric Retrospective Cohort Study (ENGOT-Cx3/CEEGOG CX2). Ann. Oncol..

[36] Lécuru F., Mathevet P., Querleu D., Leblanc E., Morice P., Daraï E., Marret H., Magaud L., Gillaizeau F., Chatellier G. (2011). Bilateral Negative Sentinel Nodes Accurately Predict Absence of Lymph Node Metastasis in Early Cervical Cancer: Results of the SENTICOL Study. J. Clin. Oncol..

[37] Mathevet P., Lécuru F., Uzan C., Boutitie F., Magaud L., Guyon F., Querleu D., Fourchotte V., Baron M., Bats A.-S. (1990). Sentinel Lymph Node Biopsy and Morbidity Outcomes in Early Cervical Cancer: Results of a Multicentre Randomised Trial (SENTICOL-2). Eur. J. Cancer Oxf. Engl..

[38] Lecuru F.R., McCormack M., Hillemanns P., Anota A., Leitao M., Mathevet P., Zweemer R., Fujiwara K., Zanagnolo V., Zahl Eriksson A.G. (2019). SENTICOL III: An International Validation Study of Sentinel Node Biopsy in Early Cervical Cancer. A GINECO, ENGOT, GCIG and Multicenter Study. Int. J. Gynecol. Cancer.

[39] Rose P.G., Bundy B.N., Watkins E.B., Thigpen J.T., Deppe G., Maiman M.A., Clarke-Pearson D.L., Insalaco S. (1999). Concurrent Cisplatin-Based Radiotherapy and Chemotherapy for Locally Advanced Cervical Cancer. N. Engl. J. Med..

[40] Morris M., Eifel P.J., Lu J., Grigsby P.W., Levenback C., Stevens R.E., Rotman M., Gershenson D.M., Mutch D.G. (1999). Pelvic Radiation with Concurrent Chemotherapy Compared with Pelvic and Para-Aortic Radiation for High-Risk Cervical Cancer. N. Engl. J. Med..

[41] Peters W.A., Liu P.Y., Barrett R.J., Stock R.J., Monk B.J., Berek J.S., Souhami L., Grigsby P., Gordon W., Alberts D.S. (2000). Concurrent Chemotherapy and Pelvic Radiation Therapy Compared with Pelvic Radiation Therapy Alone as Adjuvant Therapy after Radical Surgery in High-Risk Early-Stage Cancer of the Cervix. J. Clin. Oncol..

[42] Pötter R., Tanderup K., Schmid M.P., Jürgenliemk-Schulz I., Haie-Meder C., Fokdal L.U., Sturdza A.E., Hoskin P., Mahantshetty U., Segedin B. (2021). MRI-Guided Adaptive Brachytherapy in Locally Advanced Cervical Cancer (EMBRACE-I): A Multicentre Prospective Cohort Study. Lancet Oncol..

[43] EMBRACE. https://www.embracestudy.dk/Public/Default.aspx?embrace=embrace&main=1&sub=3&utm_source=chatgpt.com&AspxAutoDetectCookieSupport=1.

[44] University College, London (2024). A Phase III Multicentre Trial of Weekly Induction Chemotherapy Followed by Standard Chemoradiation Versus Standard Chemoradiation Alone in Patients with Locally Advanced Cervical Cancer. https://clinicaltrials.gov/study/NCT01566240.

[45] McCormack M., Eminowicz G., Gallardo D., Diez P., Farrelly L., Kent C., Hudson E., Panades M., Mathew T., Anand A. (2024). Induction Chemotherapy Followed by Standard Chemoradiotherapy versus Standard Chemoradiotherapy Alone in Patients with Locally Advanced Cervical Cancer (GCIG INTERLACE): An International, Multicentre, Randomised Phase 3 Trial. Lancet Lond. Engl..

[46] Mileshkin L.R., Moore K.N., Barnes E.H., Gebski V., Narayan K., King M.T., Bradshaw N., Lee Y.C., Diamante K., Fyles A.W. (2023). Adjuvant Chemotherapy Following Chemoradiotherapy as Primary Treatment for Locally Advanced Cervical Cancer versus Chemoradiotherapy Alone (OUTBACK): An International, Open-Label, Randomised, Phase 3 Trial. Lancet Oncol..

[47] AstraZeneca (2024). A Phase III, Randomized, Multi-Center, Double-Blind, Global Study to Determine the Efficacy and Safety of Durvalumab in Combination with and Following Chemoradiotherapy Compared to Chemoradiotherapy Alone for Treatment in Women with Locally Advanced Cervical Cancer. https://clinicaltrials.gov/study/NCT03830866.

[48] Kouhen F., Houssaini M.S. (2024). CALLA Trial: Immunotherapy in Locally Advanced Cervical Cancer. Lancet Oncol..

[49] Tewari K.S., Sill M.W., Long H.J., Penson R.T., Huang H., Ramondetta L.M., Landrum L.M., Oaknin A., Reid T.J., Leitao M.M. (2014). Improved Survival with Bevacizumab in Advanced Cervical Cancer. N. Engl. J. Med..

[50] Oaknin A., Gladieff L., Martínez-García J., Villacampa G., Takekuma M., De Giorgi U., Lindemann K., Woelber L., Colombo N., Duska L. (2024). Atezolizumab plus Bevacizumab and Chemotherapy for Metastatic, Persistent, or Recurrent Cervical Cancer (BEATcc): A Randomised, Open-Label, Phase 3 Trial. Lancet Lond. Engl..

[51] Tewari K.S., Monk B.J., Vergote I., Miller A., de Melo A.C., Kim H.-S., Kim Y.M., Lisyanskaya A., Samouëlian V., Lorusso D. (2022). Survival with Cemiplimab in Recurrent Cervical Cancer. N. Engl. J. Med..

[52] Seagen Inc. (2023). A Single Arm, Multicenter, International Trial of Tisotumab Vedotin (HuMax^®^-TF-ADC) in Previously Treated, Recurrent or Metastatic Cervical Cancer. https://clinicaltrials.gov/study/NCT03438396.

[53] Vergote I.B., Martin A.G., Fujiwara K., Kalbacher E., Bagameri A., Ghamande S., Lee J.-Y., Banerjee S., Maluf F.C., Lorusso D. (2023). LBA9 innovaTV 301/ENGOT-Cx12/GOG-3057: A Global, Randomized, Open-Label, Phase III Study of Tisotumab Vedotin vs Investigator’s Choice of Chemotherapy in 2L or 3L Recurrent or Metastatic Cervical Cancer. Ann. Oncol..

[54] Seagen A Wholly Owned Subsidiary of Pfizer (2025). A Phase 1b/2 Open-Label Trial of Tisotumab Vedotin (HuMax^®^-TF-ADC) Monotherapy and in Combination with Other Agents in Subjects with Recurrent or Stage IVB Cervical Cancer. https://clinicaltrials.gov/study/NCT03786081.

[55] Meric-Bernstam F., Makker V., Oaknin A., Oh D.-Y., Banerjee S., González-Martín A., Jung K.H., Ługowska I., Manso L., Manzano A. (2024). Efficacy and Safety of Trastuzumab Deruxtecan in Patients With HER2-Expressing Solid Tumors: Primary Results From the DESTINY-PanTumor02 Phase II Trial. J. Clin. Oncol..

[56] Gilead Sciences (2025). A Phase II Open Label Study of Sacituzumab Govitecan in Patients with Solid Tumor. https://clinicaltrials.gov/study/NCT05119907.

[57] The Project. https://www.hpv-faster-implement.eu/project/.

[58] Castle P.E., Stoler M.H., Wright T.C., Sharma A., Wright T.L., Behrens C.M. (2011). Performance of Carcinogenic Human Papillomavirus (HPV) Testing and HPV16 or HPV18 Genotyping for Cervical Cancer Screening of Women Aged 25 Years and Older: A Subanalysis of the ATHENA Study. Lancet Oncol..

[59] Latsuzbaia A., Vanden Broeck D., Van Keer S., Weyers S., Donders G., Doyen J., Tjalma W., De Sutter P., Peeters E., Vorsters A. (2022). Validation of BD Onclarity HPV Assay on Vaginal Self-Samples versus Cervical Samples Using the VALHUDES Protocol. Cancer Epidemiol. Biomark. Prev..

[60] Kowalkowska M.E., Kamińska K., Wojtysiak J., Koper K., Makarewicz A., Pietrzak B., Bomba-Opoń D., Dębska M., Wielgoś M., Grabiec M. (2025). Tumor Mutational Burden in Cervical Cancer as Potential Marker for Immunotherapy Responders. Cancers.

[61] Cao G., Yue J., Ruan Y., Han Y., Zhi Y., Lu J., Liu M., Xu X., Wang J., Gu Q. (2023). Single-cell Dissection of Cervical Cancer Reveals Key Subsets of the Tumor Immune Microenvironment. EMBO J..

[62] Oaknin A., Moore K., Meyer T., González J.L.-P., Devriese L.A., Amin A., Lao C.D., Boni V., Sharfman W.H., Park J.C. (2024). Nivolumab with or without Ipilimumab in Patients with Recurrent or Metastatic Cervical Cancer (CheckMate 358): A Phase 1–2, Open-Label, Multicohort Trial. Lancet Oncol..

[63] Jou J., Kato S., Miyashita H., Thangathurai K., Pabla S., DePietro P., Nesline M.K., Conroy J.M., Rubin E., Eskander R.N. (2023). Cancer-Immunity Marker RNA Expression Levels across Gynecologic Cancers: Implications for Immunotherapy. Mol. Cancer Ther..

[64] Liu L., Wang A., Liu X., Han S., Sun Y., Zhang J., Guo L., Zhang Y. (2022). Blocking TIGIT/CD155 Signalling Reverses CD8+ T Cell Exhaustion and Enhances the Antitumor Activity in Cervical Cancer. J. Transl. Med..

[65] Georgetown University (2025). A Phase II Trial of Combination Therapy of Pembrolizumab and Lenvatinib in Patients with Locally Advanced or Metastatic Cervical Cancer. https://clinicaltrials.gov/study/NCT04865887.

[66] Mayadev J., Zamarin D., Deng W., Lankes H.A., Pesci G., Kim H., Chino J.P., Banbury B., Sherry N., Sharon E. (2025). Neoadjuvant or Concurrent Atezolizumab with Chemoradiation for Locally Advanced Cervical Cancer: A Randomized Phase I Trial. Nat. Commun..

[67] New NRG Oncology Trial for High-Risk Locally Advanced Cervical Cancer Launching Soon!. https://www.nrgoncology.org/Home/News/Post/new-nrg-oncology-trial-for-high-risk-locally-advanced-cervical-cancer-launching-soon/.

[68] Rajawat J., Banerjee M. (2024). Poly(ADP-Ribose) Polymerase1 (PARP1) and PARP Inhibitors: New Frontiers in Cervical Cancer. Biochem. Biophys. Res. Commun..

[69] University of Oklahoma (2025). Phase II Trial of Niraparib in Combination with Dostarlimab in Patients with Recurrent or Progressive Cervix Cancer (OU-SCC-STAR). https://clinicaltrials.gov/study/NCT04068753.

[70] Saitama Medical University International Medical Center (2022). A Phase 2 Study of Pembrolizumab in Combination with Olaparib in Patients with Recurrent or Metastatic Cervical Cancer Who Had Disease Progression During or After Platinum-Based Chemotherapy. https://clinicaltrials.gov/study/NCT04641728.

[71] (2025). Daiichi Sankyo HERTHENA-PanTumor01 (U31402-277): A Phase 2, Multicenter, Multicohort, Open-Label, Proof of Concept Study of Patritumab Deruxtecan (HER3-DXd; U3-1402) in Subjects with Locally Advanced or Metastatic Solid Tumors. https://clinicaltrials.gov/study/NCT06172478.

[72] Yang L., West C.M. (2019). Hypoxia Gene Expression Signatures as Predictive Biomarkers for Personalising Radiotherapy. Br. J. Radiol..

[73] FDA Grants Breakthrough Therapy Designation of New TIL Therapy for Advanced Cervical Cancer|Center for Cancer Research. https://ccr.cancer.gov/news/article/fda-grants-breakthrough-therapy-designation-of-new-til-therapy-for-advanced-cervical-cancer.

[74] Hinrichs C. (2025). A Phase II Trial of T Cell Receptor Gene Therapy Targeting Human Papillomavirus (HPV) 16 E7 for HPV-Associated Cancers. https://clinicaltrials.gov/study/NCT05686226.

[75] Zhu Y., Zhou J., Zhu L., Hu W., Liu B., Xie L. (2022). Adoptive Tumor Infiltrating Lymphocytes Cell Therapy for Cervical Cancer. Hum. Vaccines Immunother..

[76] Mittelstadt S., Kelemen O., Admard J., Gschwind A., Koch A., Wörz S., Oberlechner E., Engler T., Bonzheim I., Staebler A. (2023). Detection of Circulating Cell-Free HPV DNA of 13 HPV Types for Patients with Cervical Cancer as Potential Biomarker to Monitor Therapy Response and to Detect Relapse. Br. J. Cancer.

[77] Valpione S., Mundra P.A., Galvani E., Campana L.G., Lorigan P., De Rosa F., Gupta A., Weightman J., Mills S., Dhomen N. (2021). Author Correction: The T Cell Receptor Repertoire of Tumor Infiltrating T Cells Is Predictive and Prognostic for Cancer Survival. Nat. Commun..

[78] Bizzarri N., Russo L., Dolciami M., Zormpas-Petridis K., Boldrini L., Querleu D., Ferrandina G., Pedone Anchora L., Gui B., Sala E. (2023). Radiomics Systematic Review in Cervical Cancer: Gynecological Oncologists’ Perspective. Int. J. Gynecol. Cancer.

[79] Han K., Zou J., Zhao Z., Baskurt Z., Zheng Y., Barnes E., Croke J., Ferguson S.E., Fyles A., Gien L. (2024). Clinical Validation of Human Papilloma Virus Circulating Tumor DNA for Early Detection of Residual Disease After Chemoradiation in Cervical Cancer. J. Clin. Oncol..

[80] (2024). Peking Union Medical College Hospital Customized Circulating Tumor DNA Testing for Cervical Cancer Recurrence Surveillance and Treatment Decisions. https://clinicaltrials.gov/study/NCT06649838.

[81] Fan J., Fu Y., Peng W., Li X., Shen Y., Guo E., Lu F., Zhou S., Liu S., Yang B. (2023). Multi-Omics Characterization of Silent and Productive HPV Integration in Cervical Cancer. Cell Genom..

[82] Lin Z., Zhou Y., Liu Z., Nie W., Cao H., Li S., Li X., Zhu L., Lin G., Ding Y. (2025). Deciphering the Tumor Immune Microenvironment: Single-Cell and Spatial Transcriptomic Insights into Cervical Cancer Fibroblasts. J. Exp. Clin. Cancer Res..

[83] Yang W., Jin X., Huang L., Jiang S., Xu J., Fu Y., Song Y., Wang X., Wang X., Yang Z. (2024). Clinical Evaluation of an Artificial Intelligence-Assisted Cytological System among Screening Strategies for a Cervical Cancer High-Risk Population. BMC Cancer.

